# Hexagonal-shaped graphene quantum plasmonic nano-antenna sensor

**DOI:** 10.1038/s41598-023-46164-2

**Published:** 2023-11-06

**Authors:** S. Kavitha, Ravi Shankar Saxena, Ashish Singh, Kamakshi Kumari, Mohammed Aneesh

**Affiliations:** 1 Department of Computer & Communication, NMAMIT (Affiliated to Nitte (Deemed to Be University)), Udupi, India; 2 Department of Electronics and Communication, GMRIT, Rajam, India; 3 Department of Electronics and Communication, University of Allahabad, Prayagraj, India; 4 Department of Electronics and Communication, Veer Bahadhur Singh Purvanchal University, Jaunpur, India

**Keywords:** Materials science, Nanoscience and technology, Optics and photonics

## Abstract

In this manuscript, a hexagonal-shaped graphene quantum plasmonic nanopatch antenna sensor is designed and investigated on silicon dioxide, zinc oxide and silicon substrates for quantum plasmonic biosensing applications. The optical properties of graphene are demonstrated using Kubo modeling to analyze the plasmon resonance characteristics of the nanopatch antenna. Nano-circuit modeling of the hexagonal-shaped graphene nano-antenna is proposed and validated using CST simulations. The parametric analysis of the hexagonal-shaped nanopatch antenna is performed using design parameters such as *R* (radius of the hexagon), *T*_*p*_ (thickness of the hexagon) and *µ*_*c*_ (chemical potential of graphene) to obtain optimum characteristics suitable for quantum plasmonic sensing applications. The study demonstrates that the proposed hexagonal-shaped nano-antenna exhibits gain of 4.9 dBi, 2.46 dBi, 14.99 dBi, 8.25 dBi, 5.15 dBi, 10.87 dBi and 2.4 dBi at 29.87 THz, 30 THz, 35 THz, 113.5 THz, 132.5 THz, 85 THz and 24 THz, respectively. The field enhancement factors observed at these frequencies are 794, 779, 584, 255, 234, 654 and 217, respectively.

## Introduction

Plasmonics is a subfield of nanophotonics involving the study of surface plasmon polaritons (SPP)^1–3^. Quantum plasmonic nano-antennas have prominent applications in biosensing since they can enhance optical energy extensively at the nanoscale, near the size of biomolecules^2, 4–6^. These devices are based on the optical properties of the metals and their plasmon resonance frequencies are dependent on the refractive index of the surrounding media^5, 7, 8^. Conventionally, quantum plasmonic nanostructures are created using noble metals^9^. The mid-infrared frequency range has shown much importance in biosensing applications since most biological materials possess vibrational frequencies in this region^10, 11^. Noble metals show plasmon resonance in the near visible region and have constraints^9^ such as low mobility, high losses, low sensitivity and tuning difficulty in the mid-infrared region^1, 12, 13^. Graphene, a two-dimensional material that produces surface plasmons within the infrared and terahertz regions, possesses extensive optical characteristics suitable to create plasmonic nano-antennas^14^ for biosensing applications. Among various nano-antenna structures, nanopatch is often used as a nanosensor since it can be easily designed and used^15^. In this regard, hexagonal-shaped graphene quantum plasmonic nano-antenna sensor is designed and studied on silicon dioxide, zinc oxide and silicon substrates.

Graphene plasmonics is an active research area that emerged recently by exploiting the unique properties of the graphene^14, 16^. Graphene has dynamically tunable plasmon resonance characteristics that can be achieved through electrostatic biasing or doping^16^. It shows low dissipation and high plasmon confinement with extended life times compared to conventional metals^10, 14^ used in the plasmonic antenna design. Moreover, graphene exhibits high quantum efficiency for quantum optical wireless communication^17^. Additionally, graphene is optically transparent, possesses high mobility, stability and it is abundantly available^18–20^. These properties of the graphene make it attractive for quantum plasmonic sensing applications^15^.

The invention of graphene in 2004 by Novoselov, stimulated the recent publication of research papers on graphene quantum plasmonic nano-antennas for sensing applications^21, 22^. Rodrigo et al. demonstrated that the tunable plasmon resonance characteristics of graphene can be exploited to implement a graphene-based plasmonic biosensor^11^. Verma et al.^23^ showed that a layer of graphene in the sensor can enhance the performance of the biosensor. The enhanced sensitivity of the sensor by including a layer of graphene in a gold-based biosensor is reported by Zeng et al.^24^. A glucose sensor using graphene transistors is developed by Zhang et al. to increase the sensitivity of the sensor^25^. Zhou et al.^26^ studied and implemented a single-layer graphene sensor for the detection of cancer molecules. A DNA sensor using six graphene transistors is demonstrated by Xu et al.^27^ to increase the sensitivity of the biosensors. These all-reported works use graphene sheet, layer or transistor on the existing biosensing devices to increase the sensitivity. The inherent property of nano-antenna, which is the high field enhancement and the extensive properties of graphene can be exploited to build a nano-antenna using graphene to increase the performance of the biosensor.

In Graphene nanomaterial carbon atoms are connected in a hexagonal lattice structure so it is compatible to design a hexagonal-shaped nano-antenna using graphene. Moreover, hexagonal shape has six vertices therefore field concentration will be more near the edges. This will enhance the gain of the nano-antenna. In this regard, hexagonal shape for the graphene nanopatch antenna is chosen. Since graphene supports hexagonal shape, sensitivity of the graphene nano-antenna sensor will improve and also leads to easy fabrication of graphene patch antenna.

Traditionally, the Drude and Lorentz models are used to describe the dispersive properties of plasmonic metals and dielectrics respectively^28–30^. But dispersive properties of the graphene are modelled using Kubo model. Kubo conductivity formula describes^28–30^ the surface conductivity of graphene, but the Drude permittivity model have to use volume conductivity of material. Since the thickness of graphene is very thin, the surface conductivity and volume conductivities supposed to be approximately same. Moreover, for the terahertz frequencies, the interband part of conductivity is negligibly small comparing with the intraband conductivity of graphene. Therefore, the surface conductivity from the Kubo model and volume conductivity from Drude model will be approximately same. The surface conductivity and plasma frequency (Drude Model) method of graphene modeling produce nearly the same simulation result for the same graphene antenna structure if they are compared. Hence dispersive properties of the graphene material is studies and analyzed using Kubo conductivity model.

In this manuscript, a hexagonal-shaped plasmonic graphene nanopatch antenna is designed and studied on different substrates for quantum biosensing biosensing applications. The nanocircuit modeling of the nanopatch antenna is proposed and validated using the finite integration technique. The next section of the paper portrays the design of a nanopatch graphene plasmonic antenna.

## Hexagonal-shaped graphene patch antenna design

**Figure 1:**
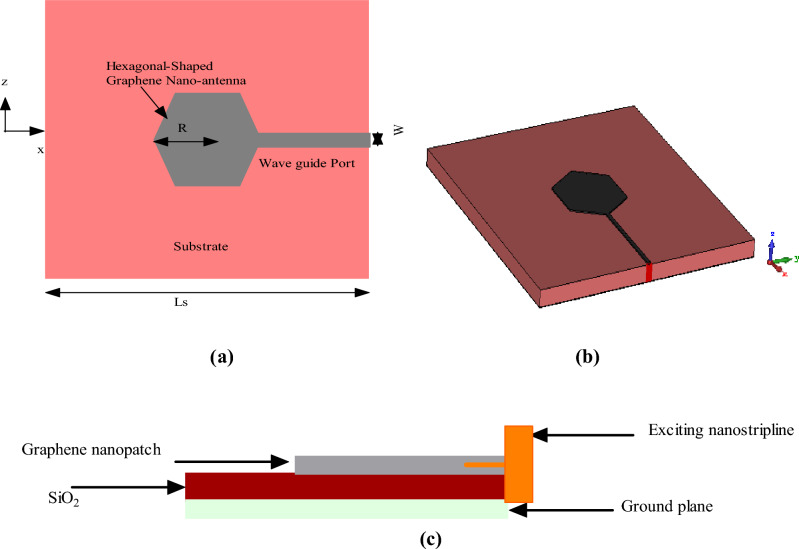
Views of hexagonal-Shaped graphene nanopatch antenna (**a**) 2D, (**b**) 3D and (**c**) side.

Figure 1a–c describe two-dimensional, three-dimensional and side views of the hexagonal-shaped graphene patch nanopatch antenna, where *R* and *T*_*p*_ are the radius and thickness of the hexagonal-shaped patch, respectively. The proposed nanostructure is fed by a waveguide port matched to 50 Ω using a nanostrip line of width *W*. The entire nanostructure is designed on a square-shaped substrate with length *L*_*s*_ = 700 nm and thickness *T*_*s*_ = 36 nm. Graphene is modeled using parameters such as temperature *T*_*g*_ = 300° K, relaxation time *t*_*r*_ = 0.1 psec, thickness *t* = 10 nm and chemical potential *µ*_*c*_ = 0.1 eV. A designed nanopatch antenna is studied in the CST Microwave Studio using silicon dioxide, zinc oxide and silicon dielectric substrates. The electromagnetic simulator used to analyze the hexagonal-shaped nanoantenna is the finite integration technique (FIT) in CST. The parametric analysis is performed on the hexagonal-shaped graphene nano-antenna to optimize the characteristics for quantum plasmonic sensing applications.

Graphene nanoptach can be produced using several methods. Micromechanical cleavage can produce millimeter sized single layer graphene sheets; Liquid-phase exfoliation method can produce graphene nanoribbons with widths less than 10 nm; using graphene oxide large dispersion-processed graphene flakes can be produced; Chemical vapor deposition can produce single and few layer graphene^19^. Even though most of these methods are decade old, because of the popularity of the graphene these methods are used to produce graphene nanostructures recently^19^.

## Dispersive properties of graphene

Antennas operating in the terahertz bands are based on dispersive materials; in this regard, the dispersive properties of graphene are analyzed in this section. At the optical frequency, material properties such as conductivity, permittivity and refractive index are complex, as expressed in Eq. (1).

1 $$\sigma_{g} \left( \omega \right) = \, \sigma_{g}^{\prime } \left( \omega \right) \, - \, j \, \sigma_{g}^{\prime \prime } \left( \omega \right)$$

*σ*_*g*_′(*ω*) and *σ*_*g*_″(*ω*) in Eq. (1) represent real and imaginary parts of graphene conductivities correspondingly. Graphene conductivity can be modeled using the Kubo conductivity model^28–30^, considering both interband and intraband conductivities of graphene, represented in Eq. (2).

2 $$\sigma_{g} \left( {\omega_{g} ,\mu_{c} , \Gamma_{g} , T_{g} } \right) = \sigma_{g\_intra} \left( {\omega_{g} ,\mu_{c} , \Gamma_{g} , T_{g} } \right) + \sigma_{g\_inter} \left( {\omega_{g} ,\mu_{c} , \Gamma_{g} , T_{g} } \right)$$

3 $$\sigma_{g\_intra} \left( {\omega_{g} ,\mu_{c} , \Gamma_{g} , T_{g} } \right) = \frac{{ - ie^{2} k_{B} T_{g} }}{{\pi \hbar^{2} \left( {\omega_{g} - i2\Gamma_{g} } \right)}}\left( { \frac{{\mu_{c} }}{{k_{B} T_{g} }} + 2\ln \left( {e^{{\frac{{\mu_{c} }}{{k_{B} T_{g} }}}} + 1} \right)} \right)$$

In comparison to the intraband conductivity, the interband conductivity of graphene can be neglected for frequencies lower than *2* ${\mu }_{c}$*/ħ* and above this limit, it can be approximated as

4 $$\sigma_{{g\_{\text{int}} er}} \left( {\omega_{g} ,\mu_{c} , \Gamma_{g} , 0} \right) = \frac{{ - ie^{2} }}{{4\pi \hbar^{2} }}\ln \left( { \frac{{2\left| {\mu_{c} } \right| - \left( {\omega_{g} - i2\Gamma_{g} } \right)\hbar }}{{2\left| {\mu_{c} } \right| + \left( {\omega_{g} - i2\Gamma_{g} } \right)\hbar }}} \right)\;\;{\text{and}}\;\;k_{B} T_{g} < < \left| {\mu_{c} } \right|,\;\;\hbar \omega_{g}$$

where ${k}_{B}$ represents Boltzman constant, *ħ* is reduced Plank’s constant, ${\mu }_{c}$ is chemical potential, ${{\omega }_{g}}$ denotes operating frequency, ${\Gamma }_{g}$ represents scattering rate, *T*_*g*_ is the temperature.

The model presented above is used to analyze the conductivity, permittivity and refractive index of graphene at the frequency of light. The permittivity ${\epsilon }_{g}\left({\omega }_{g}\right)$ and refractive index ${n}_{g}({\omega }_{g})$ are calculated using the fundamental equations given below.

5 $$\epsilon_{g} \left( {\omega_{g} } \right) = \epsilon_{gR} - j\frac{{\sigma_{g} \left( {\omega_{g} } \right)}}{{\omega_{g} \epsilon_{0} }}$$

6 $$n_{g} \left( {\omega_{g} } \right) = \sqrt {\epsilon_{g} \left( {\omega_{g} } \right)}$$

An attractive feature of graphene is its tunable conductivity. The conductivity of graphene can be easily modified with the help of chemical potential through electrostatic biasing or chemical doping. Plasmon resonance features of the graphene nanopatch antenna can be optimized using the tunability of graphene. The real part of permittivity of graphene should be negative to sustain plasmonic oscillations in graphene plasmonic nano-antenna. The imaginary part of permittivity is used to analyze the losses in the graphene at terahertz frequencies.

**Figure 2:**
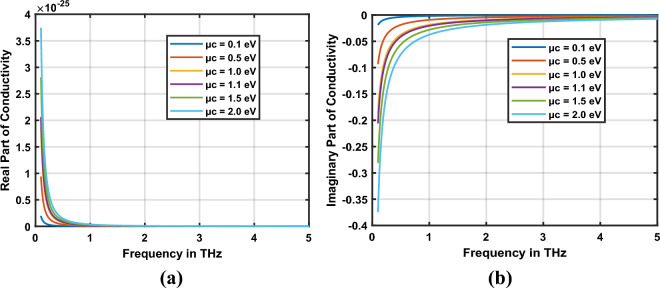
Conductivity of graphene (**a**) real part, (**b**) imaginary part.

**Figure 3:**
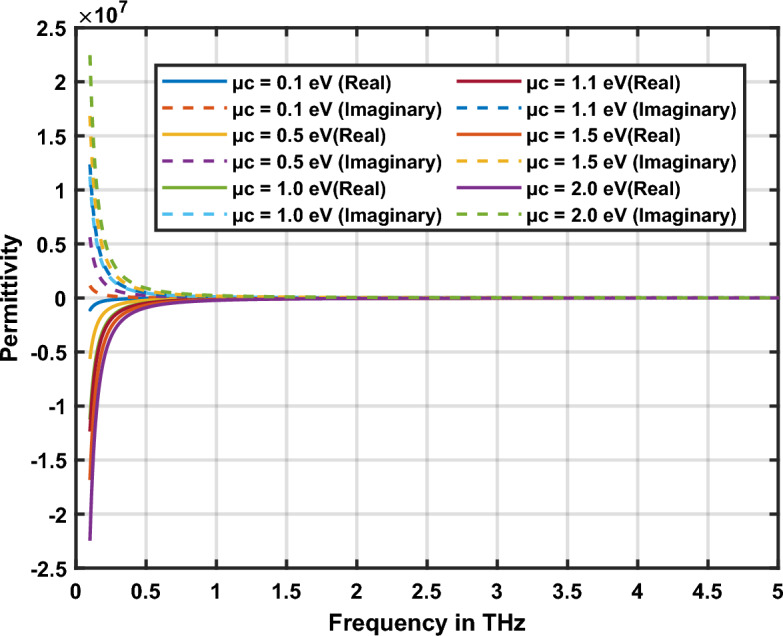
Permittivity of the graphene.

The real and imaginary components of the conductivity of graphene are depicted in Fig. 2a,b correspondingly. Graphene’s chemical potential is tuned from 0.1 to 2 eV and corresponding value of conductivity is measured. The study demonstrates that the conductivity of graphene increases at higher values of chemical potential. The complex permittivity model is derived from the conductivity of graphene and depicted in Fig. 3. Furthermore, the real component of permittivity is negative, indicating graphene is a suitable material for plasmonic antenna design. As the chemical potential rises, real permittivity of graphene becomes more negative and graphene nano-antennas can sustain high-quality plasmon oscillations at terahertz frequencies. Furthermore, losses in the nano-antenna decrease with the increase in frequency. This is indicated by the imaginary component of permittivity of graphene shown in Fig. 3. Graphene shows stable dispersive characteristics at high terahertz frequencies. These features make graphene suitable for quantum plasmonic sensing applications. When it is used as a plasmonic sensor, depending on the sensing environment, the refractive index of graphene changes, causing permittivity and conductivity changes accordingly. This causes the change in plasmon resonance characteristics of the antenna. Based on this analysis, sensing parameters can be interpreted.

The chemical potential of the graphene can be varied up to ~ 2 to 3 eV using Kubo model. At high level of chemical potential graphene loses its linear electron dispersion and spatial isotropy. This will impact the sensing performance of the graphene nanopatch antenna.Traditionally, the chemical potential tuning of the graphene is achieved by using electrostatic gate (bias) voltage or with chemical doping. The variation of the bias voltage results in change in the conductivity of graphene. In this manuscript, graphene patch antenna is designed on a substrate whose gate voltage (bias voltage) which is connected in between the substrate and graphene sheet can modify the chemical potential and thus changing conductivity^30^. Chemical doping of graphene achieved through surface transfer doping or substitutional doping^31^.

## Nano-circuit modeling of hexagonal-shaped graphene nano-antenna

Nano-circuit modeling is required to theoretically understand the behavior of nano-antennas. The concept is essential for interconnecting plasmonics with existing electronics. This will model the graphene nano-antenna using nano-inductor, nano-resistor and nano-capacitor. In this regard, nano-circuit modeling of a hexagonal-shaped graphene nanopatch antenna is designed and validated through CST simulations. Based on the fundamental concepts of nano-circuit theory, elements that exhibit negative real permittivity are modeled as nano-inductors and those that exhibit positive permittivity are modeled as nano-capacitors. The imaginary part of permittivity of the material is modeled as a nano-resistor; this signifies the ohmic losses contributed by the material. In this paper, a hexagonal graphene nanopatch is designed on silicon dioxide, zinc oxide and silicon substrates. The substrate materials exhibit positive real permittivity at terahertz frequencies and graphene exhibits negative real permittivity at the plasmon resonance. Hence, graphene nanopatch can be modeled as a nano-inductor (negative real permittivity) series with a nano-resistor (imaginary part of permittivity). The substrate materials are modeled as a nano-capacitor (positive real permittivity) and the fringing field effect is modeled using a fringing field nano-capacitor (positive background permittivity). All these aspects are considered in designing nano-circuit model for the proposed plasmonic graphene nanopatch antenna.

The impedance of an nano/optical antenna is given in Eq. (7) using potential difference *V*_*dg*_ and differential current I_*dg*_ using e^jωt^ time convention.

7 $$z_{nano\_g} = \frac{{V_{d} }}{{I_{d} }} = \frac{EL}{{j\omega_{g} \varepsilon EA}}$$

where *E, L* and *A* denote the incident field, the length of the hexagon and the cross-section area of the nanostructure, respectively. The nano-impedance of the proposed hexagonal-shaped nanostructure is derived using Eq. (7) as

8 $$z_{nano\_h} = \frac{2}{{j\omega_{g} \varepsilon 3\surd 3R}}$$

The fringing nano-impedance of the proposed hexagonal-shaped nanostructure is evaluated considering the background positive permittivity of the medium from Eq. (7) as

9 $$z_{nano\_frgh} = \frac{1}{{j\omega_{g} \varepsilon_{0} 3\surd 3R^{ } }}$$

The nano-inductance of the hexagonal-shaped nanostructure from Eq. (7) is

10 $$L_{hg} = \frac{2}{{\omega_{g}^{2} \varepsilon_{realp} 3\surd 3R}}$$

The nano-capacitance of the hexagonal-shaped nanostructure from Eq. (7) is

11 $$C_{hg} = \frac{{\varepsilon_{realp} 3\surd 3R}}{2}$$

12 $$C_{fringe\_hg} = \varepsilon_{0} 3\surd 3R$$

The nano-resistance of the hexagonal-shaped nanostructure from Eq. (7) is

13 $$R_{hg} = \frac{2}{{\omega_{g} \varepsilon_{imgp} 3\surd 3R}}$$

where $\varepsilon_{realp}$ and $\varepsilon_{imgp}$ symbolize the real and imaginary parts of the permittivity’s correspondingly. The model presented above satisfies the plasmon resonance criterion given in Eq. (14).

14 $$L_{hg} C_{hg} = \omega_{g}^{ - 2}$$

The proposed silicon dioxide based hexagonal-shaped nanopatch antenna with *R* = 110 nm has a nano-inductance of 18.538 femtoH, a nano-capacitance of 8.856 attoF and a nano-resistance of 0.02 KΩ at 30 THz. Further, a fringing nano-capacitance of 1.947 attoF is observed at the resonance.

**Figure 4:**
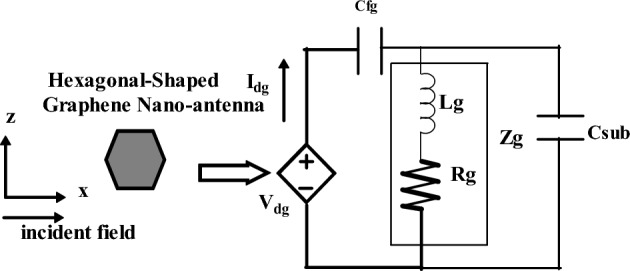
Nano-circuit model of hexagonal-Shaped graphene patch antenna.

Figure 4 depicts the nano-circuit modeling of the proposed hexagonal-shaped graphene nanopatch antenna. For the designed graphene quantum plasmonic antenna, an optical field is applied in the x direction. In Fig. 4, the fringing nano-capacitance is represented by C_fg_, the nano-impedance of the graphene nanostructure is represented by Z_g_ and the nano-capacitance of the substrate is denoted by C_sub_.

### Effects of substrates and fringinging fileds on the performnace of nanoptach antenna

The substrate capacitance changes the nano-impedance of the nano-circuit of the nano-antenna. The nano-impedance

$$\begin{aligned} Z_{g} = & R_{g} + j\omega L_{g} \;\;\;\;\;\;\;\;\;\;\;\;\;\;\;\;\;\;\;\;\;\;\;\;\;{\text{without}}\;\;{\text{C}}_{{{\text{sub}}}} \\ Z_{g} = & \left. {R_{g} + j\omega L_{g} } \right\|\frac{1}{{j\omega C_{sub} }}\;\;\;\;\;\;\;\;\;\;\;\;\;{\text{with C}}_{{{\text{sub}}}} \\ {\text{i}}.{\text{e}}.\;\;\;\;Z_{g} = & \frac{{R_{g} + j\omega L_{g} }}{{1 - \omega^{2} L_{g} C_{sub} + j\omega R_{g} C_{sub} }} \\ \end{aligned}$$

This change will shift the resonance frequency slightly towards lower frequency region and reduces the reflections in the antenna. This leads to the change in the resonance characteristics of the nano-antenna. The proposed nano-antenna is designed on the substrate so that it will couple the graphene nanoptach efficiently. This makes the enhancement and localization of the optical signal in nanopatch antenna. The fringing nano-capacitance (C_fg_) comes parallel with the nano-impedance of the nanoptach antenna.

$$\begin{aligned} Z_{g} = & R_{g} + j\omega L_{g} \;\;\;\;\;\;\;\;\;\;\;\;\;\;\;\;\;\;\;\;\;\;\;\;\;{\text{without}}\;\;{\text{C}}_{{{\text{sub}}}} \;\;{\text{and}}\;\;{\text{C}}_{{{\text{fg}}}} \\ Z_{g} = & \left. {R_{g} + j\omega L_{g} } \right\|\frac{1}{{j\omega (C_{sub} + C_{fg} )}}\;\;\;\;\;\;\;\;\;\;\;\;\;{\text{with C}}_{{{\text{sub}}}} {\text{and}}\;\;{\text{C}}_{{{\text{fg}}}} \\ {\text{i}}.{\text{e}}.\;\;\;\;Z_{g} = & \frac{{R_{g} + j\omega L_{g} }}{{1 - \omega^{2} L_{g} (C_{sub} + C_{fg} ) + j\omega R_{g} (C_{sub} + C_{fg} )}} \\ \end{aligned}$$

This change will shift the resonance frequency slightly towards low frequency region and reduces the reflections in the antenna similar to C_sub_. Due to fringing effect, patch antenna would look wider compared to its actual dimension. Near field that leaks out of the patch into the dielectric medium surrounding the conductor. This increases the field enhancement area in graphene plasmonic antenna. This increases the radiation efficiency of the nano-antenna. But due to the extended field enhancement area localization and confinement of field at the nanoscale will alter. Hence fringing fields play significant role in tuning the resonance frequency and spreading the enhanced field to a larger area.

The value of Z_g_ is dependent on C_fg_, and C_sub_. The substrate capacitance and fringing capacitance are interrelated. Substrates with higher permittivity can increase the fringing effect. The fringing field effect is also dependent on the shape of patch substrate, and ground plane design. The amount of fringing field is a function of thickness of substrate, higher the thickness greater is the fringing effect. Larger fringing fields increase effective aperture, gain and radiation efficiency but increases losses due to edge diffraction and radiation losses. The fringing fields also introduce impedance matching issues in the nano-antenna. Moreover, the resonance frequency will be shifted from the desired frequency. In addition, fringing fields can excite higher order modes so bandwidth may be reduced. Therefore, there is trade off should be considered between gain and radiation losses when we optimize the values of C_fg_, Z_g_, and C_sub_ for achieving desired antenna performance.

## Results and discussions

The proposed hexagonal-shaped graphene nanopatch antenna is studied on silicon dioxide, zinc oxide and silicon substrates. The design parameters, such as the radius of the hexagon *R*, thickness of the hexagon *T*_*p*_ and chemical potential of the graphene *µ*_*c*_ are varied to obtain the optimum plasmon resonance behavior of the graphene patch antenna.

### Silicon dioxide based hexagonal-shaped graphene nanopatch antenna 

**Figure 5:**
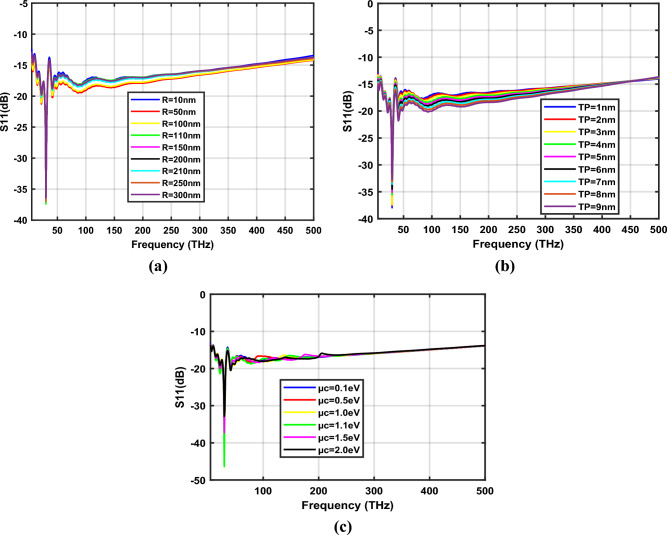
Variation of S_11_ parameters of SiO_2_ based hexagonal-shaped graphene patch antenna with (**a**) *R*, (**b**) *T*_*p*_ and (**c**) *µ*_*c*_.

Figure 5a–c depict the S_11_ parameters of the hexagonal-shaped graphene nanopatch antenna on a silicon dioxide substrate for design parameters *R*, *T*_*p*_ and *µ*_*c*_ respectively. The study demonstrates that the proposed hexagonal-shaped graphene nanopatch is resonating at 29.87 THz with a minimum reflection coefficient. The hexagonal-shaped graphene nano-antenna exhibits reflection coefficients suitable for quantum plasmonic sensing applications, i.e., below − 20 dB for frequencies in the range 20–24 THz, 27.5–32 THz and 40.5–42.5 THz when the antenna parameter *R* is changed from 10 to 300 nm. Furthermore, as the thickness of the hexagon *T*_*p*_ increases from 1 to 10 nm, reflections in the graphene nano-antenna increases. By tuning the chemical potential of graphene, the plasmon resonance characteristics of the graphene nanopatch antenna can be easily changed, as represented in Fig. 5c. The study demonstrates that, for *µ*_*c*_ = 0.5 eV and µ_*c*_ = 1.1 eV SiO_2_ based nano-antennas exhibit minimum reflection coefficients of − 41.04 dB and − 46.38 dB, correspondingly. The detailed analysis of the plasmon resonance features of the hexagonal-shaped graphene nanopatch antenna is listed in Table 1. The optimum plasmonic characteristics are observed for *R* = 110 nm, *T*_*p*_ = 3 nm and *W* = 10 nm at µ_*c*_ = 0.1 eV.

**Table 1:** Resonance frequency of SiO_2_ based hexagonal-shaped graphene nano-antenna.

*W* = 10 nm, *T*_*P*_ = 3 nm and *µ*_*c*_ = 0.1 eV			*W* = 10 nm, *R* = 110 nm and *µ*_*c*_ = 0.1 eV			*W* = 10 nm, *R* = 110 nm and *T*_*P*_ = 3 nm		
*R* (nm)	f (THz)	S_11_ (dB)	*T*_*P*_ (nm)	f (THz)	S_11_ (dB)	*µ*_*c*_ (eV)	f (THz)	S_11_ (dB)
10	29.87	− 34	1	29.87	− 37.96	0.1	29.87	− 37.41
50	29.87	− 32.94	2	29.87	− 37.52	0.5	29.87	− 41.04
100	29.87	− 33.89	3	29.87	− 37.41	1.0	30.87	− 33.09
110	29.87	− 37.41	4	29.87	− 35.55	1.1	29.87	− 46.38
150	29.87	− 36.56	5	29.87	− 35.08	1.5	29.87	− 37.28
200	29.87	− 36.17	6	29.87	− 34.50	2.0	29.87	− 32.86
210	29.87	− 35.91	7	29.87	− 33.58			
250	29.87	− 36.99	8	29.87	− 33.58			
300	29.87	− 36.44	9	29.87	− 32.69			
			10	29.87	− 32.02			

**Figure 6:**
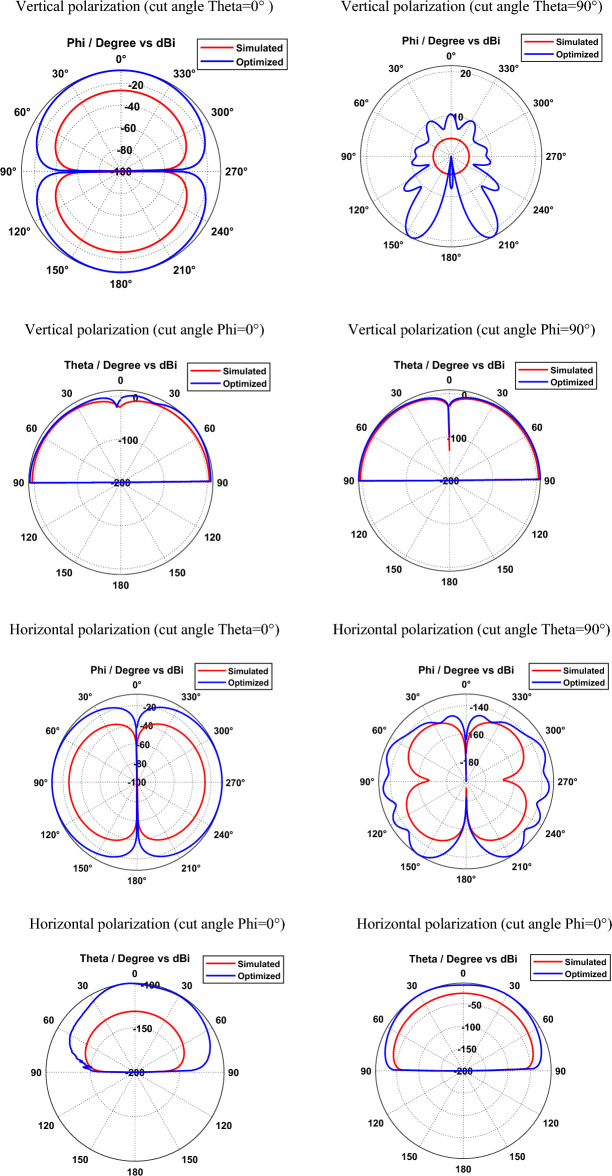
Radiation patterns of SiO_2_ based hexagonal-shaped graphene patch antenna.

Simulated and optimized field patterns of hexagonal-shaped graphene patch nano-antenna on silicon dioxide substrate for vertical and horizontal polarization with different cut angles at 29.87 THz are shown in Fig. 6. The study demonstrates that the proposed graphene nano-antenna shows suppressed back radiation for the cut angles Phi = 0° and Phi = 90° compared to cut angles Theta = 0° and Theta = 90°. The beam width for the vertically polarized SiO_2_ based graphene antenna is 89.9°, 17.6°, 35° and 43.7° in the directions Theta = 0°, Theta = 90°, Phi = 0° and Phi = 90° respectively. Furthermore, horizontally polarized graphene nano-antenna exhibits beam widths of 89.9°, 18.7°, 68.6° and 37° in the directions Theta = 0°, Theta = 90°, Phi = 0° and Phi = 90° respectively. The study demonstrates that vertically and horizontally polarized SiO_2_ based graphene nano-antenna produces directional radiation in Phi = 0° and Phi = 90° directions.

### Zinc oxide based hexagonal-shaped graphene nanopatch antenna 

Plasmon resonance characteristics of the proposed zinc oxide-based hexagonal-shaped nano-antenna with *W* = 10 nm and *W* = 20 nm is discussed in this section.

#### Zinc oxide based hexagonal-shaped graphene nanopatch antenna with W = 10 nm

**Figure 7:**
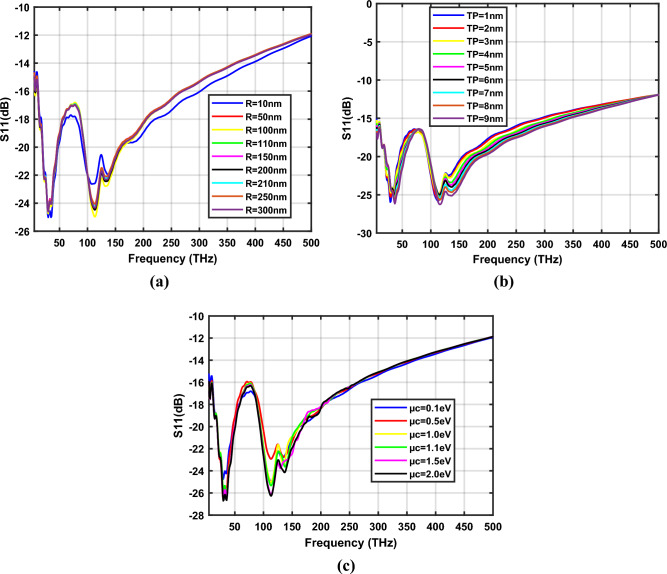
Variation of S_11_ parameters of ZnO based hexagonal-shaped graphene patch antenna (*W* = 10 nm) with (**a**) *R*, (**b**) *T*_*p*_ and (**c**) *µ*_*c*_.

S_11_ coefficients of a *W* = 10 nm hexagonal-shaped graphene nanopatch antenna on a zinc oxide substrate for design parameters *R*, *T*_*p*_ and *µ*_*c*_ are depicted in Fig. 7a–c respectively. The study demonstrates that the proposed hexagonal-shaped graphene nano-antenna resonates at 30 THz, 35 THz, 113.5 THz and 132.5 THz with minimum reflection at *W* = 10 nm, as the design parameters vary. The frequency bands showing reflection coefficients below − 20 dB are 19–46 THz, 91–157 THz when *R* is varied from 10 to 300 nm, 21 to 55 THz, 94 to 185 THz when *T*_*p*_ is changed from 1 to 10 nm and 19 to 46 THz, 93 to 167 THz when *µ*_*c*_ is changed from 0.1 to 2.0 eV. The detailed analysis is tabulated in Table 2. Optimum characteristics of the hexagon-shaped zinc oxide-based graphene nano-antenna are observed for *R* = 100 nm and *T*_*P* =_ 3 nm at *µ*_*c*_ = 0.1 eV.

**Table 2:** Resonance frequency of ZnO based hexagonal-shaped graphene nano-antenna (*W* = 10 nm).

*W* = 10 nm, *T*_*P*_ = 3 nm and *µ*_*c*_ = 0.1 eV			*W* = 10 nm, *R* = 100 nm and *µ*_*c*_ = 0.1 eV			*W* = 10 nm, *R* = 100 nm and *T*_*P*_ = 3 nm		
*R* (nm)	f (THz)	S_11_ (dB)	*T*_*P*_ (nm)	f (THz)	S_11_ (dB)	*µ*_*c*_ (eV)	f (THz)	S_11_ (dB)
10	30	− 25.01	1	29	− 25.96	0.1	30	− 24.75
	35	− 25.1		32	− 24.622		35	− 24.25
	111.5	− 22.60		112.5	− 25.80		113.5	− 24.97
	136.5	− 21.95		131.5	− 22.46		132.5	− 22.78
50	30	− 24.42	2	30	− 25.23	0.5	30	− 26.65
	35	− 23.86		35	− 24.40		35	− 25.79
	113.5	− 24.13		113.5	− 23.63		113.5	− 22.94
	132.5	− 22.13		131.5	− 22.70		136.5	− 22.72
100	30	− 24.75	3	30	− 24.75	1.0	30	− 25.69
	35	− 24.25		35	− 24.25		36	− 26.20
	113.5	− 24.97		113.5	− 24.97		112.5	− 25.04
	132.5	− 22.78		132.5	− 22.78		135.5	− 23.39
110	30	− 24.56	4	30	− 24.40	1.1	30	− 25.71
	35	− 24.04		36	− 24.70		36	26.26
	113.5	− 24.39		114.5	− 25.10		113.5	− 25.33
	132.5	− 22.35		136.5	− 23.39		137.5	− 23.64
150	30	− 24.42	5	31	− 24.34	1.5	30	− 26.49
	35	− 23.90		36	− 24.94		36	− 26.31
	113.5	− 24.07		114.5	− 24.96		112.5	− 26.13
	133.5	− 22.12		136.5	− 23.71		129.5	− 23.78
200	30	− 24.53	6	31	− 24.26	2.0	30	− 26.71
	35	− 24.07		37	− 25.15		36	− 26.65
	113.5	− 24.47		114.5	− 24.98		113.45	− 26.66
	132.5	− 22.44		136.5	− 23.98		136.5	− 24.14
210	30	− 24.49	7	31	− 24.53			
	35	− 24.02		37	− 25.57			
	113.5	− 24.14		115.5	− 25.36			
	134.5	− 22.22		136.5	− 24.42			
250	30	− 24.56	8	37	− 25.80			
	35	− 23.96		115.5	− 25.66			
	113.5	− 24.05		136.5	− 24.67			
	133.5	− 22.08						
300	30	− 24.61	9	37	− 26.17			
	35	− 24.07		116.5	− 26.26			
	113.5	− 24.35		136.5	− 25.13			
	132.5	− 22.31						
			10	35	− 25.65			
				37	− 26.49			
				116.5	− 26.97			
				136.5	− 25.59			

**Figure 8:**
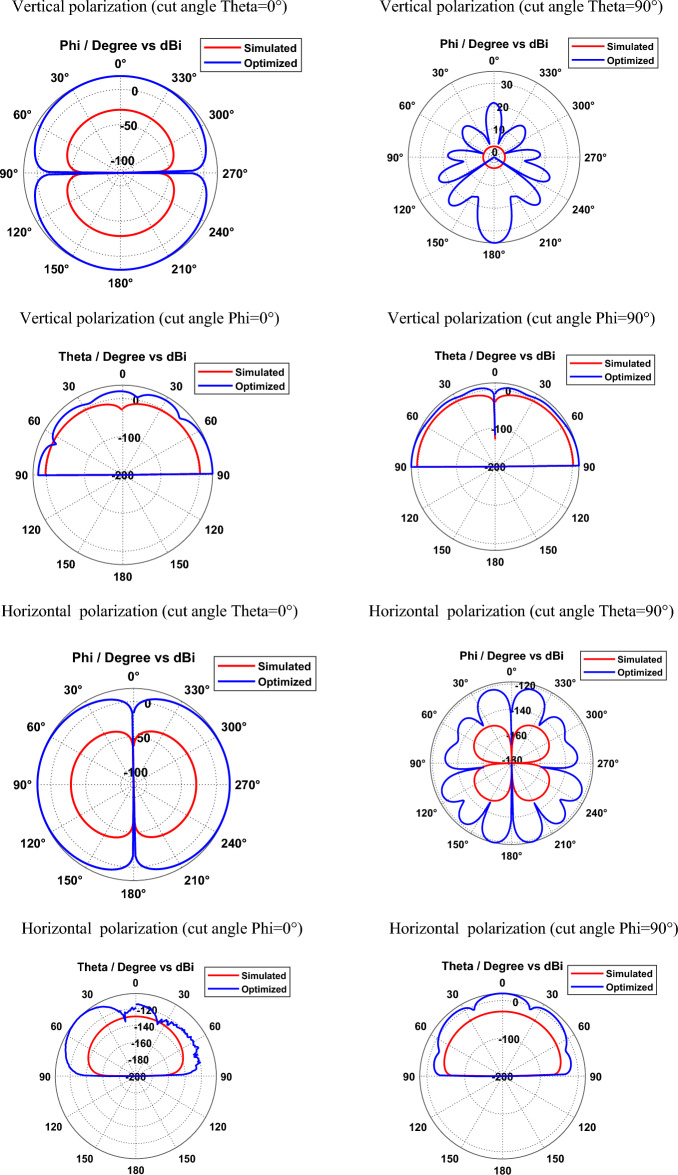
Radiation patterns of ZnO based hexagonal-shaped graphene patch antenna (W = 10 nm) at 30 THz.

**Figure 9:**
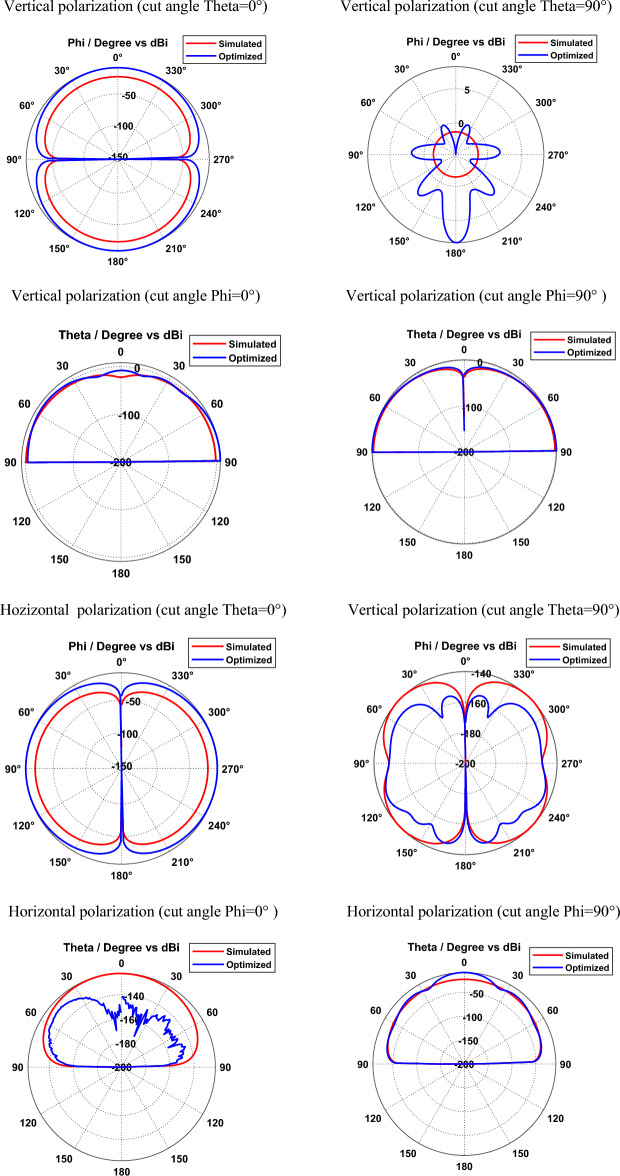
Radiation patterns of ZnO based hexagonal-shaped graphene patch antenna (*W* = *10* nm) at 113.5 THz.

Figures 8 and 9 depict the simulated and optimized field patterns of the hexagonal-shaped graphene nanopatch antenna on a zinc oxide substrate for vertical and horizontal polarization with different cut angles at 30 THz and 115 THz respectively. The study demonstrates that the proposed graphene nanopatch antenna shows low back radiation for cut angles Phi = 0° and Phi = 90° for vertical and horizontal polarization. A vertically polarized ZnO based graphene antenna exhibits half-power beam widths of 89.9°, 18.1°, 24.2° and 22.7° in the directions Theta = 0°, Theta = 90°, Phi = 0° and Phi = 90° respectively, at 30 THz. Furthermore, horizontally polarized graphene nano-antenna shows beam widths of 89.9°, 14.2°, 29.9° and 20.9° in the directions Theta = 0°, Theta = 90°, Phi = 0° and Phi = 90° respectively at 30 THz. The study demonstrates that vertically and horizontally polarized ZnO based graphene nano-antenna produces low beam width patterns in the Theta = 90° direction for vertical and horizontal polarization at 30 THz.

At 113.5 THz, a vertically polarized ZnO based graphene antenna shows half-power beam widths of 89.9°, 21.7°, 26.5° and 34.8° in the direction Theta = 0°, Theta = 90°, Phi = 0° and Phi = 90° respectively and a horizontally polarized ZnO based graphene antenna shows half-power beam widths of 89.9°, 24.8°, 27.8° and 22.4° in the direction Theta = 0°, Theta = 90°, Phi = 0° and Phi = 90° respectively.

#### Zinc oxide based hexagonal-shaped graphene nanopatch antenna with W = 20 nm

**Figure 10:**
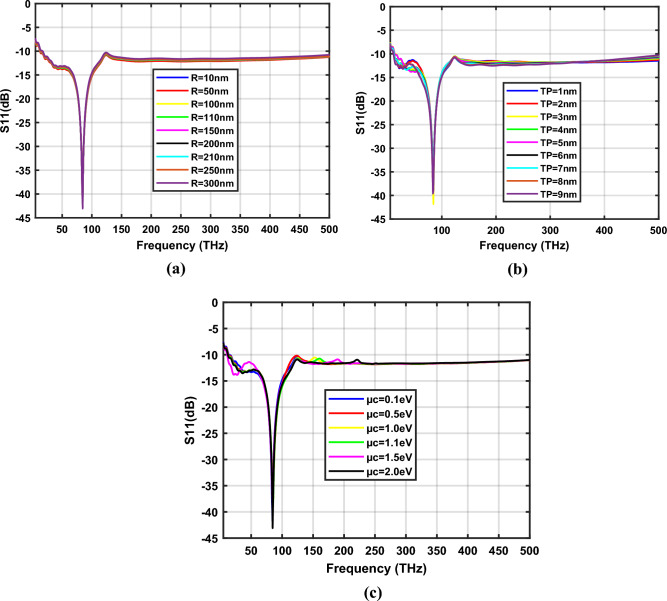
Variation of S_11_ parameters of ZnO based hexagonal-shaped graphene patch antenna (*W* = 20 nm) with (**a**) *R*, (**b**) *T*_*p*_ and (**c**) *µ*_*c*_.

Fig. 10a–c depict the S_11_ parameters of the zinc oxide-based hexagon-shaped graphene nano-antenna with *W* = 20 nm for design parameters *R*, *T*_*p*_ and *µ*_*c*_ respectively. The study demonstrates that hexagonal-shaped nano-antenna exhibits reflection coefficients below − 20 dB for frequencies ranging from 70 to 91 THz, 74 to 92 THz and 77 to 93 THz when the design parameters *R, T*_*p*_ and *µ*_*c*_ are varied, respectively. Furthermore, the proposed hexagonal-shaped graphene nano-antenna shows − 41.84 dB reflection at 85 THz for *R*=100 nm, *T*_*p*_ = 3 nm and *µ*_*c*_ = 0.1 eV on a zinc oxide substrate. The detailed analysis of the proposed nano-antenna is tabulated in Table 3.

**Table 3:** Resonance frequency of ZnO based hexagonal-shaped graphene nano-antenna (*W* = 20 nm).

*W* = 20 nm, *T*_*P*_ = 3 nm and *µ*_*c*_ = 0.1 eV			*W* = 20 nm, *R* = 100 nm and *µ*_*c*_ = 0.1 eV			*W* = 20 nm, *R* = 100 nm and *T*_*P*_ = 3 nm		
*R* (nm)	f (THz)	S_11_ (dB)	*T*_*P*_ (nm)	f (THz)	S_11_ (dB)	*µ*_*c*_ (eV)	f (THz)	S_11_ (dB)
10	85	− 40.87	1	85	− 35.38	0.1	85	− 41.84
50	85	− 36.33	2	85	− 34.57	0.5	85	− 36.29
100	85	− 41.84	3	85	− 41.84	1.0	85	− 39.33
110	85	− 38.96	4	85	− 37.62	1.1	85	− 39.91
150	85	− 40.13	5	85	37.17	1.5	85	− 39.18
200	85	− 42.11	6	84	− 33.52	2.0	85	− 43.09
210	85	− 40.31	7	84	− 33.86			
250	85	− 40.84	8	84	− 39			
300	85	− 43.75	9	84	− 39.62			
			10	85	− 40.84			

**Figure 11:**
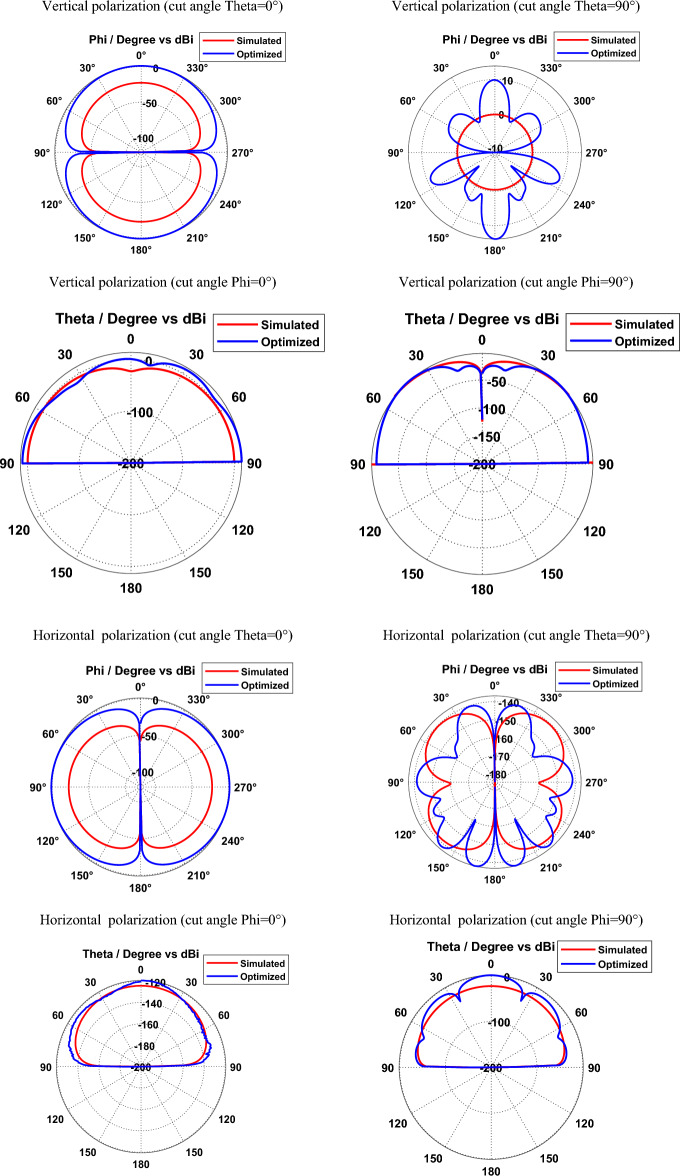
Radiation patterns of ZnO based hexagonal-shaped graphene patch antenna (W = 20 nm).

Simulated and optimized field patterns of the hexagonal-shaped graphene nanopatch antenna on a zinc oxide substrate with *W* = 20 nm for vertical and horizontal polarization for different cut angles at 85 THz are shown in Fig. 11. The study demonstrates that proposed graphene nano-antenna has low back lobes for cut angles Phi = 0° and Phi = 90° compared to cut angles Theta = 0° and Theta = 90°. The beam width for the vertically polarized zinc oxide-based graphene antenna with *W* = 20 nm is 89.9°, 15.2°, 22.4° and 59.6° in the directions Theta = 0°, Theta = 90°, Phi = 0° and Phi = 90° respectively. Furthermore, horizontally polarized zinc oxide-based graphene antennas exhibit beam widths of 89.9°, 15.4°, 32.5° and 21° in the directions Theta = 0°, Theta = 90°, Phi = 0° and Phi = 90° respectively. The study demonstrates that vertically and horizontally polarized ZnO based graphene nano-antenna produces directional radiation in Phi = 0° and Phi = 90° directions.

### Silicon based hexagonal-shaped graphene nanopatch antenna 

**Figure 12:**
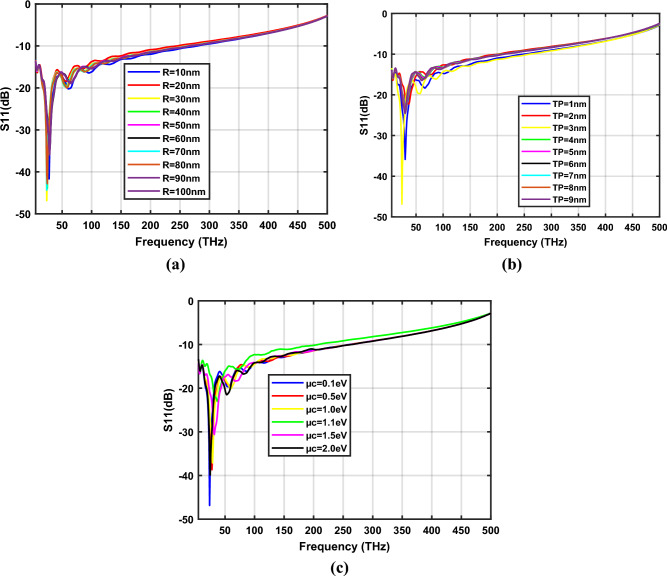
Variation of S_11_ parameters of Si based hexagonal-shaped graphene patch antenna with (**a**) *R*, (**b**) *T*_*p*_ and (**c**)*µ*_*c*_.

Figure 12a–c represent the reflection coefficients of the hexagonal-shaped graphene nanopatch antenna on a silicon substrate for the design parameters *R*, *T*_*p*_ and *µ*_*c*_ respectively. The study demonstrates that a silicon-based graphene nanopatch antenna resonates from 24 to 29 THz when the design parameter *R* is varied from 10 to 100 nm. Further, it is resonating from 24 to 31 THz for the antenna parameter; *T*_*p*_, spanned from 1 to 10 nm and when *µ*_*c*_ is changed from 0.1 to 2 eV it resonates from 24 to 37 THz. The frequency bands from 17 to 37 THz, 17 to 40 THz and 18 to 43 THz exhibit reflection coefficients below − 20 dB for the variation of design parameters *R*, *T*_*p*_ and *µ*_*c*_ respectively. Furthermore, at 2 eV chemical potential, hexagonal-shaped graphene nano-antenna is resonating at 25 THz and 54 THz with reflection coefficients of − 39.88 dB and − 21.45 dB, respectively. The optimum characteristics of the hexagonal-shaped nanopatch antenna are observed at *R* = 30 nm, *T*_*p*_ = 3 nm and *µ*_*c*_ = 0.1 eV with a − 46.86 dB reflection coefficient at 24 THz. A detailed analysis of the hexagonal-shaped graphene nanopatch antenna on a silicon substrate is given in Table 4.

**Table 4:** Resonance frequency of Si based hexagonal-shaped graphene nano-antenna.

*W* = 10 nm, *T*_*P*_ = 3 nm and *µ*_*c*_ = 0.1 eV			*W* = 10 nm, *R* = 30 nm and *µ*_*c*_ = 0.1 eV			*W* = 10 nm, *R* = 30 nm and *T*_*P*_ = 3 nm		
*R* (nm)	f (THz)	S_11_ (dB)	*T*_*P*_ (nm)	f (THz)	S_11_ (dB)	*µ*_*c*_ (eV)	f (THz)	S_11_ (dB)
10	28	− 41.68	1	30	− 35.88	0.1	24	− 46.86
20	25	− 30.68	2	37	− 22.25	0.5	28	− 38.68
30	24	− 46.86	3	24	− 46.86	1.0	29	− 37.10
40	25	− 43.90	4	31	− 24.02	1.1	37	− 23.01
50	29	− 33.85	5	31	− 24.21	1.5	32	− 30.63
60	29	− 35.26	6	30	− 24.53	2.0	23	− 39.88
70	24	− 44.34	7	30	− 24.63			
80	25	− 42.81	8	30	− 24.61			
90	29	− 35.93	9	30	− 24.53			
100	29	− 35.59	10	30	− 24.41			

**Figure 13:**
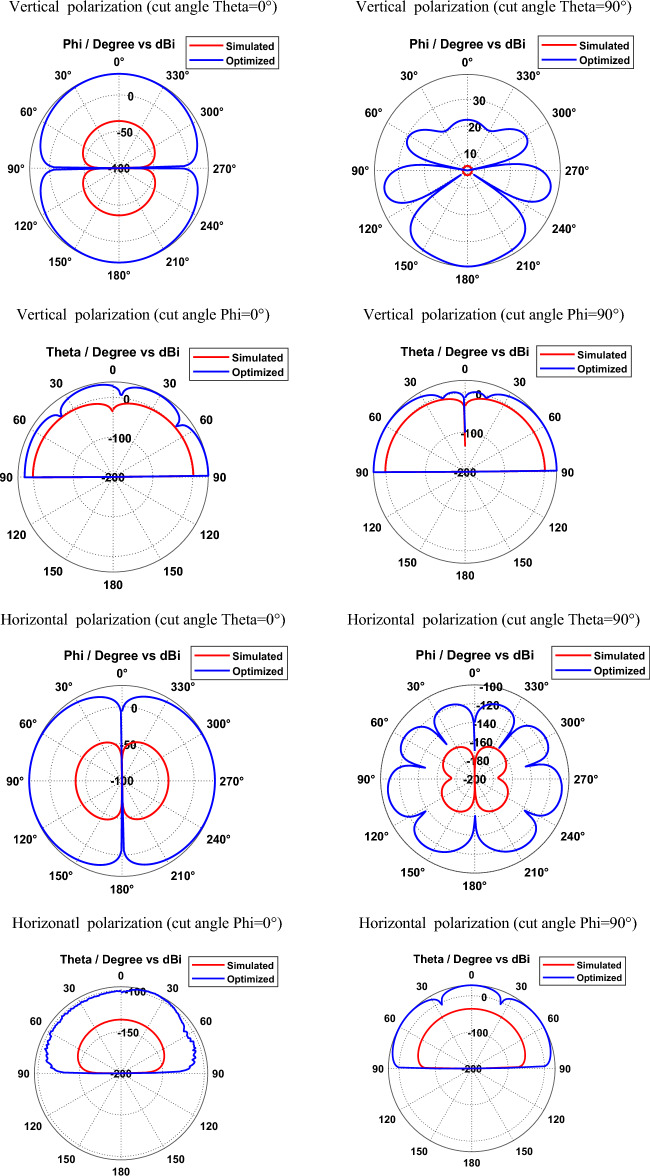
Radiation patterns of Si based hexagonal-shaped graphene patch antenna.

Figure 13 depicts the simulated and optimized field patterns of the vertically and horizontally polarized hexagonal-shaped graphene patch nano-antenna on a silicon substrate for different cut angles at 24 THz. The study demonstrates that a vertically polarized Si based graphene antenna exhibits half-power beam widths of 89.9°, 87.1°, 21.0 and 42.1° in the directions Theta = 0°, Theta = 90°, Phi = 0° and Phi = 90° respectively. Furthermore, horizontally polarized graphene nano-antenna shows beam widths of 89.9°, 21.5°, 18.1° and 32.4° in the directions Theta = 0°, Theta = 90°, Phi = 0° and Phi = 90° respectively on a silicon substrate.

### Validation of nano-circuit modeling of hexagonal-shaped graphene nanopatch antenna

**Figure 14:**
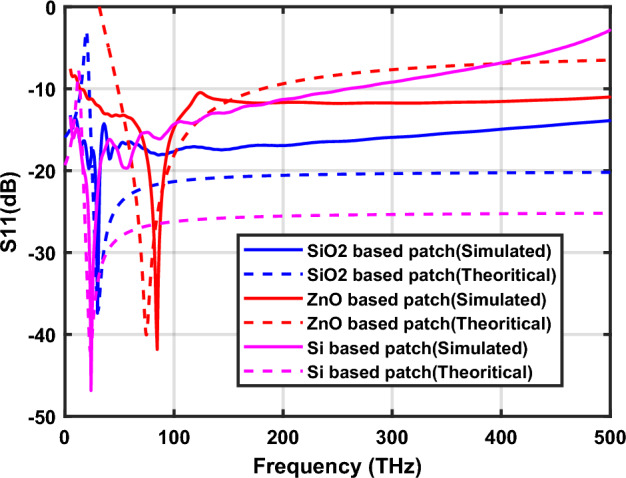
Validation of nano-circuit modeling of hexagonal-shaped graphene patch antenna.

The validation of the proposed nano-circuit modeling of hexagonal-shaped graphene nanopatch antenna is performed by comparing it with CST simulation. Initially, the S parameters of the nano-antenna are calculated using the nano-impedance of the hexagonal-shaped graphene antenna. Then a graph of the S parameter as a function of frequency is plotted. The CST-simulated and theoretical S_11_ characteristics of hexagonal-shaped graphene nanopatch antenna are depicted in Fig. 14. The study demonstrates that theoretical and CST simulations are almost match each other, demonstrating the validation of the proposed model.

The optimum characteristics of the proposed hexagonal-shaped graphene nanopatch antenna on silicon dioxide, zinc oxide and silicon dieletric substrates are shown in Table 5. The designed graphene antenna exhibits badwidths of 6 THz, 27 THz, 72 THz, 15 THz and 13 THz on silicon dioxide, zinc oxide (*W* = 10 nm, first resonance), zinc oxide (*W* = 10 nm, second resonance), zinc oxide (*W* = 20 nm) and silicon substrates, respectively. The hexagonal-shaped graphene nano-antenna has a gain of 4.9 dBi, 2.46 dBi, 14.99 dBi, 8.25 dBi, 5.15 dBi, 10.87 dBi and 2.4 dBi at 29.87 THz, 30 THz, 35 THz, 113.5 THz, 132.5 THz, 85 THz and 24 THz, respectively . The study demonstrates that a hexagonal-shaped graphene nanoptach antenna exhibits minimum reflection and high gain on a silicon substrate. Further, graphene nano-antenna on a zinc oxide substrate has more bandwidth compared to other substrates. The field enhancement factors are calculated using the formula E_0_/Ei, where E_i_ is the incident field and E_0_ is the output enhanced field. It is observed that a maximum enhancement factor of 794 is obtained for graphene nanoptach on SiO_2_ substrate. This feature of nano-antenna can improve the sensitivity of the biosensor in biomedical applications.

**Table 5:** Optimum characteristics of hexagonal-shaped graphene nano-antenna.

Substrate	F (THz)	S_11_ (dB)	Bandwidth (THz)	Gain (dBi)	Field enhancement (E_0_) (V/m)	Field enhancement factor
SiO_2_	29.87	− 37.41	6	4.9	7.13 × 10^9^	794
ZnO (*W* = 10 nm)	30	− 24.75	27	2.46	7.78 × 10^9^	779
	35	− 24.25		14.99	5.83 × 10^9^	584
	113.5	− 24.97	72	8.25	2.54 × 10^9^	255
	132.5	− 22.78		5.15	2.33 × 10^9^	234
ZnO (*W* = 20 nm)	85	− 41.84	15	10.87	6.53 × 10^9^	654
Si	24	− 46.86	13	2.4	6.49 × 10^9^	217

## Conclusion

A hexagonal-shaped graphene nanopatch antenna is designed and analyzed on silicon dioxide, zinc oxide and silicon dielectric substrates. Dispersive properties of graphene, such as permittivity and conductivity are studied using the Kubo model. The study demonstrates that graphene exhibits negative permittivity to support plasmon resonance at terahertz frequency. The nano-circuit model for the hexagonal-shaped nanopatch antenna is proposed and validated using CST simulations. The detailed analysis of the proposed hexagonal-shaped nanoptach antenna is performed using antenna parameters *R *(radius of the hexagon), *T*_*p*_ (thickness of the hexagon) and *µ*_*c*_ (chemical potential of graphene). The study demonstrates that the proposed hexagonal-shaped graphene nano-antenna exhibits minimum reflection and high gain on a silicon substrate. Further, the graphene nanopatch antenna on a zinc oxide substrate has more bandwidth compared to other substrates. The optimum characteristics of the graphene nanopatch antenna occur at 29.87 THz with − 37.41 dB, 30 THz with − 24.75 dB, 35 THz with − 24.25 dB, 113.5 THz with − 22.78 dB, 132.5 THz with − 22.78 dB, 85 THz with − 41.84 dB and 24 THz with − 46.86 on silicon dioxide, zinc oxide and silicon substrates, which are appropriate for quantum plasmonic biosensing applications.

## Data Availability

The datasets used and/or analyzed during the current study available from the corresponding author on reasonable request.
